# Treatment of stage IIIb/IV non-small cell lung cancer with Pemetrexed plus Oxaliplatin after failure of Erlotinib as second-line treatment

**DOI:** 10.1007/s12032-013-0550-7

**Published:** 2013-04-11

**Authors:** Sheng-Bin Shi, Rong-Hang Hu, Jie-Lin Qi, Xiao-Yong Tang, Jing Tian, Rui Li, Chun-Xiao Chang

**Affiliations:** 1 Department of Internal Medicine, Shan Dong Tumor Hospital, Jinan, 250117 People’s Republic of China; 2 Department of Thoracic Surgery, Affiliated Hospital of Jining Medical University, Jining, People’s Republic of China

**Keywords:** Lung adenocarcinoma, Oxaliplatin, Pemetrexed, Erlotinib as second-line treatment

## Abstract

To determine the efficacy and toxicity of Pemetrexed plus Oxaliplatin in patients suffering from stage IIIb or IV lung adenocarcinoma and being treated with Erlotinib as second-line treatment, a total of 45 patients were randomly divided into two groups. One group was treated with 500 mg/m^2^ Pemetrexed plus 100 mg/m^2^ Oxaliplatin, and the other was treated with 500 mg/m^2^ Pemetrexed plus 75 mg/m^2^ Cisplatin. All drugs were administered on day one of a 21-day cycle. In the Oxaliplatin group, 3 patients (13.6 %) experienced partial response (PR), 9 patients (41.0 %) showed stable disease (SD), and 10 patients (45.5 %) had progressive disease (PD). In the Cisplatin group, 2 patients (8.7 %) experienced PR, 7 patients (30.4 %) showed SD, and 14 patients (60.9 %) had PD. The PFS of the Oxaliplatin group and the Cisplatin group was 4.45 months (95 % CI 4.10–4.80) and 3.96 months (95 % CI 3.68–4.24) (*P* = 0.03), respectively. The median overall survival (OS) was 10.8 months (95 % CI 10.2–11.5) and 10.7 months (95 % CI 10.2–11.3) (*P* = 0.72), respectively. There was no statistically significant difference in the occurrence rate of grades 3 and 4 myelotoxicity between the two groups. However, there was a significant difference in the occurrence rate of grades 3 and 4 gastrointestinal reactions and peripheral neurotoxicity between the two groups (*P* < 0.05). A regime combining Pemetrexed and Oxaliplatin was marginally effective and well tolerated in patients with stage IIIb or IV lung adenocarcinoma who have received Erlotinib as second-line treatment.

## Introduction

Lung cancer is one of the major malignant diseases that threaten human health and is the leading cause of death among all cancer patients [1]. Non-small cell lung cancer (NSCLC) accounts for about 80 % of all forms of lung cancer. Over two-third of NSCLC cases are diagnosed in the advanced stage [2]. Currently, comprehensive treatments, including chemotherapy and targeted drug therapies, are the major therapies for advanced NSCLC. Doublet chemotherapies consisting of platinum plus one of the third-generation agents have become the standard regime [3]. Different platinum-based chemotherapy doublets, which have similar levels of efficacy, are the first-line chemotherapies for patients with good performance [4]. Consequently, the current treatment guidelines recommend 4–6 cycles of chemotherapy. For the second-line treatment, EGFR–TKI monotherapy tended to be more effective in East Asian patients in terms of PFS and ORR compared with standard second-line chemotherapy and was associated with less toxicity and better tolerability [5–7]. However, it is unclear how to treat patients with disease progression or drug resistance after targeted therapy, which has become a hot research field.

Pemetrexed is an antifolate agent with multiple targets. It inhibits the activities of several enzymes that are involved in purine and pyrimidine synthesis. In elderly NSCLC patients, promising results have been obtained when Pemetrexed is used in both first-line and maintenance therapies. For example, the efficacy and safety of Pemetrexed treatment have been evaluated in elderly patients with advanced non-squamous NSCLC and a performance status of 0–1. Data from large-scale randomized studies have been retrospectively analyzed for patients aged younger than 65 and older than 65. And it has been shown that the safety and efficacy are similar between two groups [8].

Oxaliplatin, also named diaminocyclohexane oxalatoplatinum, is a third-generation platinum analogue that inhibits DNA replication. It belongs to a distinct family of platinum compounds [9]. Several randomized studies have suggested that Oxaliplatin-based doublets exert anti-tumor activities that are as effective as those of Cisplatin- or Carboplatin-based regimes [10, 11]. In a phase III study, the efficacy and tolerability of Gemcitabine and Oxaliplatin (GEMOX) with Paclitaxel and Carboplatin (PCb) in chemotherapy-naive patients with stage IIIb/IV NSCLCs have been compared, and it has been shown that the PFS, OS, and objective response rate of GEMOX are similar to those of PCb [12]. In a one experience, the combination of Pemetrexed, Oxaliplatin and Bevacizumab has been well tolerated and has promising activity as a first-line therapy in random patients with stage IV non-squamous NSCLC. The objective response rate is 55.3 % (95 % CI 39.5–71.1). The median PFS and OS are 6.2 (95 % CI 5.4–9.0) and 14.6 (95 % CI 9.8–19.5) months, respectively [13]. Misset et al. [14] have shown that the combination of Pemetrexed and Oxaliplatin can be administered every 21 days using full therapeutic doses of each agent with acceptable tolerability and that the recommended dose for phase II studies is 500 mg/m^2^ Pemetrexed plus 120 mg/m^2^ Oxaliplatin in patients suffering from metastatic solid tumors. The toxicity profile of Oxaliplatin, particularly when compared with Cisplatin, makes it an alternative treatment for patients unable to tolerate Cisplatin [15]. In this study, we evaluated the efficacy and safety of Pemetrexed plus Oxaliplatin and compared those with Pemetrexed plus Cisplatin in patients who have previously received Erlotinib.

## Materials and methods

### Patients

Eligible patients included those who were histologically or cytologically diagnosed and confirmed to have locally advanced or metastatic lung adenocarcinoma, and failed to respond to Erlotinib as second-line treatment between October 2009 and September 2011 at the Shandong Tumor Hospital. The other inclusion criteria were the following: age 18–75 years; ECOG performance status (PS) 0–2; adequate hematological and hepatic function and renal function; life expectancy of at least 12 weeks; and at least one measurable lesion according to the modified response evaluation criteria in solid tumors (RECIST). Exclusion criteria included active infection, uncontrolled cardiac disease, progressive brain metastases, uncontrolled pleural effusions, prior systemic treatment with Pemetrexed, and pregnancy or breastfeeding. This study was conducted with the approval of the ethics committee of the tumor hospital in Shandong Province. All patients provided written informed consent before the study. The patients were randomly divided into Oxaliplatin group and Cisplatin group by random number table. There were no significant differences in gender, smoking history, pathological type, cancer stage, ECOG score, and chemotherapy regime and cycle between the two groups at first visit. Distribution between the groups was balanced (*P* > 0.05) (Table 1).

**Table 1:** Baseline characteristics of the overall population (*n* = 45)

Factors	Oxaliplatin group	Cisplatin group	*P*
Median age (range)	66.5 (48–73)	61.5 (49–72)	
Gender			0.67
Male	10 (45.5 %)	9 (39.1 %)	
Female	12 (54.5 %)	14 (60.9 %)	
Performance status			0.66
0	11 (50.0 %)	10 (43.5 %)	
1	11 (50.0 %)	13 (56.5 %)	
Stage			0.32
IIIb	4 (18.2 %)	6 (26.1 %)	
IV	18 (81.8 %)	17 (73.9 %)	
Smoking status			0.60
Smoker	14 (63.6 %)	16 (69.6 %)	
Non-smoker	8 (36.4 %)	7 (30.4 %)	
First-line regimen			0.15
Gemcitabine–cisplatin	9 (40.9 %)	11 (47.8 %)	
Gemcitabine–carboplatin	10 (45.5 %)	8 (34.8 %)	
Vinorelbine–cisplatin	3 (13.6 %)	4 (17.4 %)	

### Treatment

Patients received 500 mg/m^2^ Pemetrexed plus 120 mg/m^2^ Oxaliplatin or 75 mg/m^2^ Cisplatin. All drugs were given on day one of each of the 21-day cycle. Each patient received a daily dose of 1 mg oral folic acid from 1–2 weeks before the first dose of Pemetrexed to 3 weeks after the treatment was discontinued. Intramuscular injections of 1 mg vitamin B12 were administered at least 7 days before the first dose of Pemetrexed and were repeated once every 9 weeks. All patients were pre-medicated with 4 mg dexamethasone the day before, on the day of, and the day after chemotherapy. Treatment was continued for up to 6 cycles unless disease progressed, or unacceptable toxicity became apparent, or the patient no longer wished to continue the treatment.

### Evaluation and statistical methods

Tumor responses of the patients were assessed using CT according to the Response Evaluation Criteria in Solid Tumors (RECIST, ver. 1.0) after two cycles of chemotherapy. According to the RECIST guideline, complete response (CR), PR, SD, and PD were determined. Adverse events were assessed according to the Common Terminology Criteria for Adverse Events (CTCAE) version 3.0. PFS was counted from the day of initial treatment to the day of documentation of disease progression or death, and OS was measured from the day of initial treatment till death or the last follow-up treatment. PFS and overall survival curves were constructed using the Kaplan–Meier method.

## Results

### Patient characteristics

A total of 45 patients were enrolled in this study. All patients were suffering from lung adenocarcinoma and had progressed during Erlotinib maintenance therapy. In the Oxaliplatin group, 68.2 % of the patients were over the age of 65; while in the Cisplatin group, only 43.5 % of the patients were over 65 years old.

### Response

**Fig. 1:**
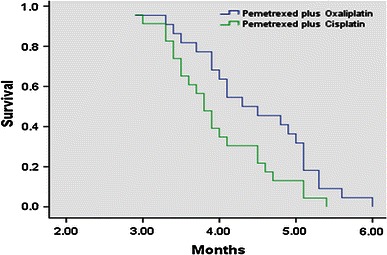
Progression-free survival in the study population (*P* = 0.03)

**Fig. 2:**
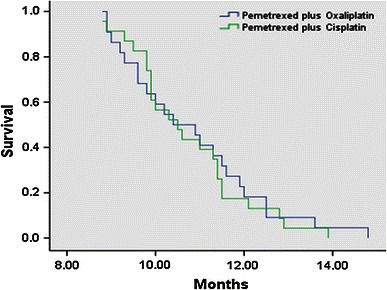
Overall survival in the study population (*P* = 0.72)

In the Oxaliplatin group, 22 patients were evaluable for response. Three patients (13.6 %) experienced a partial response (PR), 9 patients (41.0 %) showed stable disease (SD), and 10 patients (45.5 %) had progressive disease (PD). In the Cisplatin group, 23 patients were evaluable for response. Two patients (8.7 %) experienced a PR, 7 patients (30.4 %) showed SD, and 14 patients (60.9 %) had PD. The PFS of the Oxaliplatin group and the Cisplatin group was 4.45 months (95 % CI 4.10–4.80) and 3.96 months (95 % CI 3.68–4.24) (*P* = 0.03), respectively. The median overall survival (OS) was 10.8 months (95 % CI 10.2–11.5) and 10.7 months (95 % CI 10.2–11.3) (*P* = 0.72), respectively. The response rate was 13.6 and 8.7 %, respectively (Table 2; Figs. 1, 2).

**Table 2:** Efficacy evaluation of Pemetrexed plus Oxaliplatin or Cisplatin

Responses	Oxaliplatin group	Cisplatin group	*P*
Partial response	3 (13.6 %)	2 (8.7 %)	0.67
Stable disease	9 (41.0 %)	7 (30.4 %)	0.46
Progressive disease	10 (45.5 %)	14 (60.9 %)	0.30
Disease control rate	12 (54.5 %)	9 (39.1 %)	0.37
Time to progression, median (95 % CI)	4.45 (4.10–4.80)	3.96 (3.68–4.24)	0.03
Overall survival, median (95 % CI)	10.8 (10.2–11.5)	10.7 (10.2–11.3)	0.72

### Toxicity

A total of 134 cycles were administered in this trial, with a median of 3 cycles per patient (range 2–5 cycles). At the time of final analysis, all patients had discontinued treatment. The most common reasons for discontinuation were disease progression (40 patients, 88.9 %), grade 4 leukocytopenia (4 patients, 8.9 %, the fourth cycle occurred), and patient refusal (1 patient, 2.2 %, the third cycle refused). All patients completed at least two cycles of treatment. No patient was dropped out of the group during the study. There were no significant differences in the occurrence rates of grades 3 and 4 hematological toxicities between the two groups. Non-hematological toxicities included diarrhea, nausea, vomiting, stomatitis, neuropathy, and constipation. The incidence of grade 3/4 nausea and vomiting was 9.1 and 26.1 % (*P* = 0.04), respectively. The occurrence rate of peripheral neurotoxicity in the Oxaliplatin group was significantly higher than that of the Cisplatin group (*P* = 0.03) (Table 3).

**Table 3:** Toxicity profile of Pemetrexed plus Oxaliplatin

Toxicity	Oxaliplatin group grade 3/4	Cisplatin group grade 3/4	*P*
Leukocytopenia	3 (13.6 %)	5 (21.7 %)	0.70
Neutropenia	3 (13.6 %)	2 (8.7 %)	0.67
Thrombocytopenia	1 (4.5 %)	2 (8.7 %)	1.0
Anemia	2 (9.1 %)	3 (13.0 %)	1.0
Diarrhea	1 (4.5 %)	1 (4.3 %)	1.0
Nausea/vomiting	2 (9.1 %)	6 (26.1 %)	0.04
Stomatitis	0	1 (4.3 %)	1.0
Neuropathy	4 (18.2 %)	0	0.03
Constipation	0	1 (4.3 %)	1.0

## Discussion

At present, lung cancer is still one of the major deadly diseases. Although new therapeutics keep emerging, the 5-year survival rate is only 8–15 % [16], which forces us to continue exploring new treatment method. One of the hot research spots is personalized therapy for lung cancer patients. The first-line treatment for lung adenocarcinoma is generally chemotherapy involving Pemetrexed; while for patient with EGFR mutation, small molecular TKI therapy is recommended. For NSCLC, the most commonly used first-line treatment is platinum-based doublet chemotherapy, such as TC, GP, and NP, which have similar efficacy [17].

A retrospective analysis of a randomized phase III trial comparing Pemetrexed with Docetaxel has shown that there are significant associations between the histological type of a patient and the efficacy outcome of Pemetrexed treatment. The benefit of the drug seems to be confined to patients with non-squamous histology [18]. Therefore, only patients with adenocarcinoma were chosen in this study. The efficacy was statistically comparable between patients in the two groups. However, the PFSs of the two groups were significantly different, which were likely because that some patients developed resistance to Cisplatin, but were still sensitive to Oxaliplatin. The OS might be impacted by treatment regime afterward. As a third-generation platinum analogue, Oxaliplatin has a low chance of causing resistance, and therefore has a similar anti-tumor efficacy with other platinum compounds [19]. Although the efficacy was similar between the two groups, we observed a higher efficacy in older patients treated with Pemetrexed plus Oxaliplatin. Whether age plays a role in the efficacy of these drugs need to be further investigated with a larger sample size.

A phase II study in advanced NSCLC patients who have previously been treated with Oxaliplatin, Pemetrexed, and Bevacizumab has shown that nine (27 %) patients have PR, 15 (44 %) patients have SD, and 10 (29 %) patients have PD. The median PFS is 5.8 months (95 % CI 4.1–7.8 months), and the median OS is 12.5 months (95 % CI 7.3–17 months) for the treatment group [20]. Scagliotti et al. [21] have conducted a phase II trial comparing Pemetrexed treatment combined with either Oxaliplatin or Carboplatin in patients with advanced NSCLC. The results have shown that the RR for Pemetrexed–Oxaliplatin and Pemetrexed–Carboplatin treatment is 26.8 and 31.6 %, respectively; and the OS is 10.5 months for both groups. The differences in the results of efficacy and PFS between the two studies may be due to the usage of first-line therapies. Charles et al. have shown that the objective response rates (complete or partial) of Gemcitabine–Oxaliplatin in first-line treatment are 15.2 %, the PFS is 4.44 months, and the OS is 9.90 months [12]. A phase II trial has evaluated a second-line therapy with Pemetrexed and Bevacizumab in 48 patients who have progressed disease after platinum-based chemotherapy. There are 5 patients with PRs (10 %) and 19 patients with SD. The median PFS and OS are 4.1 and 8.6 months, respectively [22].

In our study, there was no difference in the occurrence rate of hematological toxicity between the two groups. However, Cisplatin had gastrointestinal toxicity. Pemetrexed and Oxaliplatin have been used in a study to treat patients with advanced solid tumors, and the most common toxicity is grade 3–4 leukopenia that emerges in 17 (47 %) patients [15]. In a first-line treatment using Pemetrexed combined with Oxaliplatin, neutropenia is the most prevalent toxicity that occurs in 7.3 % of the patients, and 73 % of the patients have experienced some forms of neurotoxicity [23]. In another study, a combination of Pemetrexed, Oxaliplatin, and Bevacizumab have been used as the first-line treatment in patients with stage IV NSCLCs, and severe hematological toxicities including grade 4 neutropenia, grade 3 anemia, and grade 3 thrombocytopenia are observed (*n* = 2 each, 5.3 %) [13].

In conclusion, Pemetrexed plus Oxaliplatin was well tolerated in patients who had received Erlotinib as second-line treatment. Based on the safety profile and clinical activity, Pemetrexed plus Oxaliplatin needs to be further investigated under the same setting. This study was limited by its small sample size, and more evidence-based medicines are needed to further improve the study.
